# Hair metal concentrations in a mother-newborn population from a polluted megacity: an indicator of prenatal metal exposure

**DOI:** 10.1038/s41370-026-00869-4

**Published:** 2026-04-11

**Authors:** Christian D. Ortiz-Robles, Marvin Paz-Sabillón, Ángel Barrera-Hernández, Luz del Carmen Sánchez-Peña, Martín Noe Rangel-Calvillo, Luz María Del Razo, Betzabet Quintanilla-Vega

**Affiliations:** 1 Department of Toxicology, Center for Research and Advanced Studies (Cinvestav), Mexico City, Mexico; 2 Hospital “Dr. José María Rodríguez”, Ecatepec de Morelos, Mexico State Mexico

**Keywords:** Metals, Hair, Biomarker, Prenatal exposure

## Abstract

**Background:**

Essential elements, such as calcium (Ca), copper (Cu), and zinc (Zn) are critical for fetal development, while metals or metalloids, such as arsenic (As), antimony (Sb), boron (B), cadmium (Cd), mercury (Hg), lead (Pb), uranium (U), and vanadium (V) are potentially toxic metals (PTM) that interfere with vital processes. There is a need for biomonitoring essential and toxic metals during the uterine stage, and hair metal content may be a good biomarker.

**Objective:**

This study aimed to quantify the concentrations of 11 elements, including both essential and PTM, in hair samples of 96 newborn-mother pairs from an urban polluted area to assess fetal metal transfer.

**Methods:**

Essential elements and PTM were quantified by inductively coupled plasma mass spectrometry (ICP-MS) in hair samples. Relationships between maternal and child hair metal concentrations were examined using Spearman’s rank correlation analysis, adjusting for potential confounding variables.

**Results:**

The mothers’ hair PTM concentrations ranged from 8.0 ng/g to 7.0 µg/g in the following order: Cd <As <Sb<U <Hg<V<Pb <B, while in newborns’ hair ranged from 1.0 ng/g to 10.0 µg/g as follows: U <Cd <As <V <Sb <Hg <Pb <B. Most PTM concentrations were higher in the mothers except for B and Sb. Significant positive correlations between the mothers’ and newborns’ hair concentrations of Hg (0.7365), As (0.6987), B (0.4980), Zn (0.3786), Pb (0.3012), U (0.2691), and Ca (0.2467) were observed. In addition, the Principal Component Analysis (PCA) performed in the mother-newborn pairs to identify possible common sources of exposure showed four principal components, which were not always similar in both groups.

**Significance:**

These results suggest that PTM are transferred to the fetus, although not all showed similar relationships with their mothers' concentrations, and that hair is a reliable biomarker for assessing prenatal metal exposure.

**Impact Statement:**

The importance of knowing the exposure to PTM during the intrauterine stage is critical to prevent adverse effects in newborns and in childhood, and lies in having good biomarkers of the exposure. Hair metal concentrations in newborns could be a valuable biomarker for prenatal exposure.

## Introduction

Essential elements, such as calcium (Ca), copper (Cu), and zinc (Zn) have a significant impact on the health of pregnant women (reduces the risk of complications, including anemia, hypertension, and preeclampsia), on the development of the fetus, as well as on the health of the newborn (avoid postnatal complications) [1, 2]. These metals are necessary due to their participation in biological processes, including signaling, enzyme regulation, structural role of proteins, skeletal and the antioxidant system [3]. However, these functions can be altered by the presence of potentially toxic metals or metalloids (PTM), such as arsenic (As), antimony (Sb), boron (B), cadmium (Cd), mercury (Hg), lead (Pb), uranium (U), and vanadium (V), among others, through the displacement of essential elements from enzymes and proteins and by an imbalance in the antioxidant system [4].

Exposure to PTM during pregnancy has been associated with adverse birth outcomes, including low birth weight, preterm birth, and small for gestational age, and has a significant impact on neurodevelopment and cognitive abilities in early childhood, potentially leading to long-term health adverse effects in adulthood, such as respiratory, neurological, cardiovascular, and reproductive diseases [5–9]. Therefore, it is important to monitor prenatal exposure to PTM using indicators that reflect prolonged exposure. Hair is an excellent sample matrix to evaluate the concentrations of several environmental contaminants to reflect chronic exposures (during the last 4 to 5 months of pregnancy); its sampling is easy and non-invasive, and the samples do not require preservation until quantification [10]. In this sense, the evaluation of PTM in hair has been widely used in biomonitoring environmental exposure to metals, including children [11–14].

The municipality of Ecatepec in the Northeast of the Metropolitan Area of Mexico City (MAMC) is one of the most populated and polluted areas in the country due to high industrial activity and vehicular traffic [15]. The presence of some PTM in the air and related adverse effects in children from this area have been reported [16]. Thus, this study aimed to determine the concentrations of essential elements and PTM in hair samples as a possible biomarker of transfer from mothers to newborns (prenatal exposure) in this area, evaluating the relationship between both.

## Materials and methods

### Study design and study population

This was a cross-sectional study performed in a population from Ecatepec de Morelos, State of Mexico, located in the MAMC. The participants were women who gave birth from August to October 2015 in the Hospital “Dr. José María Rodríguez”. The inclusion criteria were mothers who were residents of MAMC Northeast (at least one year) and whose newborns were born at term (>37 gestation weeks). Mothers who were smokers, anemic, had chronic diseases, or dyed hair were not included. Ninety-six women-newborn pairs were finally included. We explained the objectives of the study, and those who agreed to participate and signed the letter of informed consent were enrolled in the study and answered the questionnaire. The study had the approval of the Ethics Committee of the Hospital and the Institutional Bioethics Committee for Research in Humans (COBISH-Cinvestav-027/2015).

### Collection of hair samples

The sampling was conducted over a period no longer than 48 h after birth, in a clean place, wearing gloves and stainless-steel scissors to cut about 0.1 to 0.15 g from newborns’ and mothers’ hair. Maternal hair was cut as close to the scalp as possible at the back of the head to minimize the inclusion of external contamination. In the case of long hair, about 3 cm posterior to the root was retained. Neonatal hair was prioritized, getting it from the back of the head or from any other part in case there was not enough quantity. The samples were stored in clearly labeled and clean plastic bags until analysis. Both maternal and neonatal hair were dry at the time of collection.

### Preparation of hair samples

To remove dust, shampoo, and hair products, the hair samples were washed beforehand. Samples were placed in weighing trays and rinsed with deionized water for 10 min, then a 0.1% Triton X-100 solution (Sigma- Aldrich, St. Louis, MO, USA) was added by constantly moving for 20 min and rinsed 5 times with deionized water. Finally, the samples were wrapped in absorbent paper (wipers) [17]. The use of Triton X-100 to eliminate external contamination from hair without affecting the internal metal content was previously validated [18, 19].

After the washing process, an aliquot of 25 mg of each hair sample was weighed and placed in Teflon vessels for microwave wet digestion: 3 mL of nitric acid (65% v/v suprapur® HNO_3_), 300 µL of hydrogen peroxide (30% suprapur® H_2_O_2_) (both obtained from Merck Millipore, Darmstadt, Germany) and 1 mL of deionized water were added, and vessels were closed and placed in a microwave digester (ETHOS ONE, Milestone Srl., Italy) to 1500 watts and 150 °C for 25 min. After this time, the vessels were allowed to cool, and the digested samples were placed in polypropylene tubes and diluted to 10 mL of deionized water. The calibration graph and hair reference material were subject to the same digestion treatment.

### Quantification of metals in hair samples by ICP-MS

Metals in digested hair samples were quantified in a NexION 300D ICP-MS spectrometer (Perkin Elmer, MA, USA). More details of the determination are given in Table S1. Trace elements were evaluated in samples diluted at 1:10, while macro elements were diluted at 1:100. The equipment was conditioned before any analysis according to the supplier’s optimization specifications, using the NexION Setup Solution, Multielement standards: std 2, std 3, std 4, std 5, and Hg were purchased from Perkin Elmer, Pure Plus (Norwalk, CT, USA). The signals of the low, medium, and high mass elements were evaluated: beryllium (Be)-9 m/z > 4000 counts per second (cps), (indium) In-115 m/z > 45,000 cps, and uranium (U)-238 m/z > 40,000 cps with a relative standard deviation (RSD) < 0.03%, background < 1 cps, and a suitable ratio of cesium oxide (CeO)/cesium (Ce) and Ce + +/Ce. The calibration graphs were prepared using six-point multi-element standards (Perkin Elmer, Pure Plus). The cps values of each element issued by NexION’s Syngistix software were processed using the Excel software, determining the equation of the graph (6 points) and calculating the concentrations of the elements in the samples, considering the dilution and weight of the sample. The correlation coefficient of the graph was also calculated and defined as acceptable at >0.995.

The quantification of metals/metalloids was performed in the Toxicology Laboratory of Research and Service (LISTO) of Cinvestav, which is accredited by the Mexican Accreditation Entity (ema A.C.) with the number INV-007-013/19.

The method validation included the detection and quantification limits, the range within curves, precision, linearity, repeatability, reproducibility, bias, and selectivity. The human hair reference material (NCS DC73347a) used for validation was obtained from NCS Testing Technology Co. (Beijing, China) and was routinely used in all analyses. The accuracy obtained was 100 ± 20% and the RSD < 15%. For external quality control, the Cinvestav laboratory participated in the Quebec Multielement External Quality Assessment Scheme (QMEQAS) for multielements in hair, coordinated by the Quebec Center for Toxicology. The global accuracy obtained was 94.21% (*n* = 27), and the correlation coefficients obtained in three hair samples (QM-H-Q1707, QM-H-Q1716, and QM-H-Q1725) were 0.9967 for As, 0.9779 for Cd, 0.9939 for Cu, 0.9999 for Hg, 0.9357 for Pb, 0.9913 for Sb, 0.9626 for U, and 0.9455 for Zn.

### Statistical analysis

The distribution of variables was assessed using the Shapiro-Wilk test. Since most element concentrations exhibited non-normal distributions (*p* < 0.05), data were log-transformed to meet normality assumptions. Descriptive univariate analyses were performed, and central tendency values (median and 25th and 75th percentile values) and intervals were presented. To determine the relationship of hair metal concentrations between mothers and their children and the relationship between hair essential elements and PTM concentrations in newborns or mothers, partial correlation analyses were conducted by Spearman’s rank correlation test, after adjusting for potential confounders (maternal age, socioeconomic status, passive smoking, frequent use of lead-glazed ceramics, and living near an industry). Partial correlation coefficients (rho) were then calculated between the residuals of maternal and newborn concentrations. As a supplementary analysis, these residual-based correlations were also estimated considering the frequency of use of lead-glazed ceramics, socioeconomic status, passive smoking, living near an industry, and frequent seafood consumption, excluding the stratification variable from the adjustment model. Differences in hair metal concentrations between mothers and newborns, in relation to the consumption of non-nutritive substances and vitamins, maternal habits, and potential sources of metal exposure near the maternal residence, were evaluated using the Mann–Whitney U test or the Kruskal–Wallis test, as appropriate. The principal component analysis (PCA) was performed separately on maternal and newborn metal concentration datasets to identify potential common sources among the elements. PCA with octagonal rotation was performed and the number of components to retain was determined based on two criteria: (i) eigenvalues greater than 1 (Kaiser criterion) and (ii) visual inspection of the scree plot for the identification of the inflection point (Fig. S1) and those elements with loading factors greater than 0.4 were considered significant for interpretation of the components [20]. In both the newborn and maternal datasets, four principal components were retained, accounting for 58.85% and 60.94% of the total variance, respectively (Fig. S2). StataCorp LLC version 15.1 was used for all analyses (Stata Corp, College Station, TX, USA), and *p* < 0.05 was considered statistically significant.

## Results

### General characteristics of the population

The general characteristics of mother-newborn pairs are shown in Table 1. The mothers’ median age was 22 years old, 33% of them were primiparas, and all had singleton pregnancies. Overcrowding, defined as more than two individuals sharing a sleeping room, was present in 41.6% of mothers’ households, reflecting a low socioeconomic status. The newborns had the following characteristics: 39 weeks of age, 3076.1 g of weight, 50 cm of height, and 34.9 cm of head circumference. Most newborns (99%) had an APGAR value of 9.

**Table 1:** Sociodemographic and clinical characteristics of maternal and newborn participants.

Characteristic (*n* = 96)	Median (IQR)	*n* (%)
**Maternal**		
Age (years)	22.7 (21.9–23.4)	
Number of births		
1 birth		32 (33.3)
2–4 births		64 (67.3)
Socioeconomic status*		
Low		40 (41.6)
Not-low		56 (58.4)
**Newborn**		
Gestational age (weeks)	39.2 (39.0–39.4)	
Birth weight (g)	3076.1 (3005.8–3148.0)	
Height (cm)	50 (49.4–50.5)	
Head circumference (cm)	34.9 (34.5–35.4)	
APGAR score at 5 minutes:		
8		1 (1.0)
9		95 (99)

*IQR* Interquartile range. *Based on overcrowding, more than two individuals sharing a sleeping room.

### Mothers’ habits and potential contributors to exposure

The mothers’ habits and potential contributors to exposure during pregnancy are shown in Table 2; these were obtained through the questionnaire. The mothers’ main habits were consumption of vitamins and minerals (95.8%), non-nutritive substances (17.7%) and frequent seafood (47.9%), having dental amalgams (14.6%), use of lead-glazed ceramics (30.0%), and being passive smokers (35.4%). Other potential contributors to exposure were having repair shops at home (11.4%) or living near blacksmiths (19.7%), shoe repairing shops (8.3%), tire repairing shops (10.4%), or vehicle repair shops (26.0%), industries (18.7%), or a family member with an employment related to some source of metal exposure (42.7%). Detailed data are available in the supplementary material (Tables S2–S4).

**Table 2:** Mother's habits and potential contributors to metal exposure.

Characteristic	Number of mothers	Percentage
Consumption of vitamins and minerals		
Yes	92	95.8
No	6	4.2
Consumption of non-nutritive substances		
Yes	17	17.7
No	79	82.3
Frequent consumption of seafood		
Yes	46	47.9
No	50	52.1
Use of dental amalgams		
Yes	14	14.6
No	80	85.4
Frequent use of lead-glazed ceramics		
Yes	29	30.0
No	67	70.0
Passive smoker		
Yes	34	35.4
No	62	64.6
Shops at home		
Yes	11	11.4
No	85	88.6
Blacksmith shop near the home		
Yes	19	19.7
No	77	80.3
Shoe repair shop near the home		
Yes	8	8.3
No	88	91.7
Tires repair shop near the home		
Yes	10	10.4
No	86	89.6
Vehicle repair shop near the home		
Yes	25	26.0
No	71	74.0
Industry		
Yes	18	18.7
No	76	79.2
Family member with employment related to a source of exposure		
Yes	41	42.7
No	55	57.3

### Hair metal concentrations in mothers and newborns

**Fig. 1:**
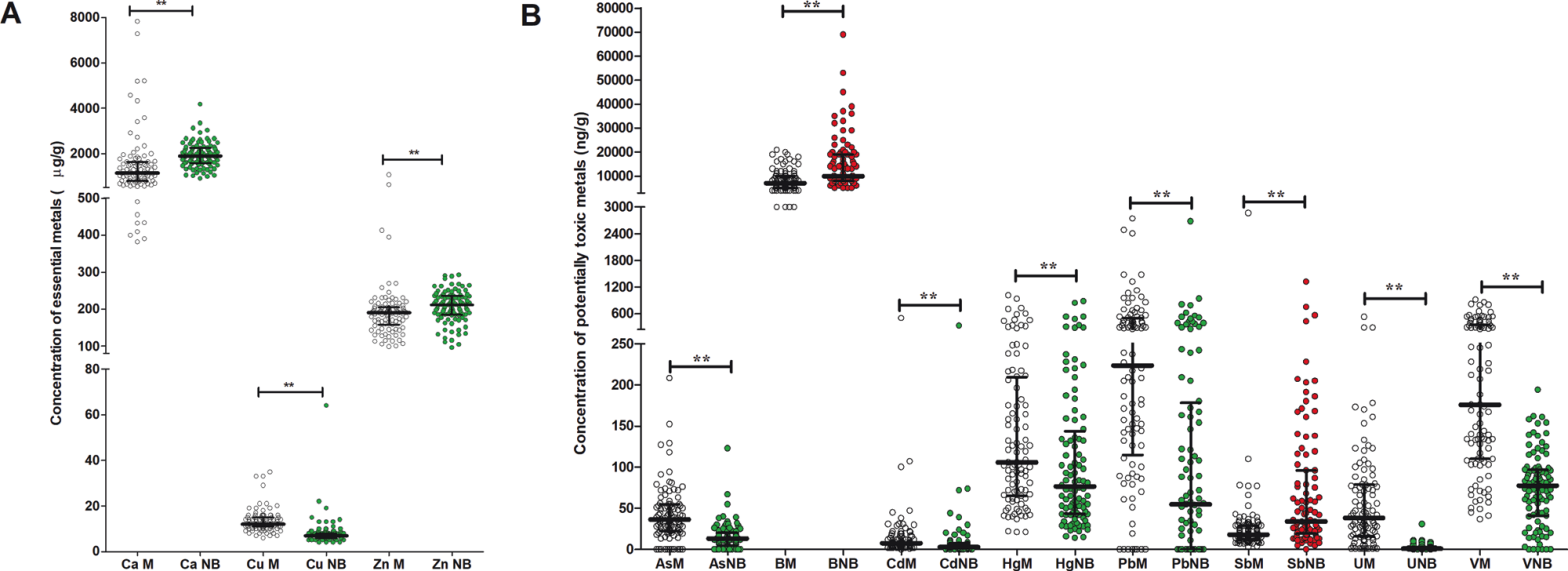
Concentrations of essential and potentially toxic metals. **A** Essential metals and **B** Potentially toxic metals. Data are presented as median and IQR. Uncolored circles represent the metal concentration for each mother (M) and green circles represent the metal concentration for each newborn (NB) when it was lower than their mother´s, and red circles represent the metal concentration for each NB when it was higher than their mother’s, *n* = 96, ** *p* < 0.01.

Eleven elements (Ca, Cu, Zn, B, As, Cd, Hg, Sb, Pb, U, and V) were quantified, and the concentrations of essential elements and PTM in hair samples from mothers and newborns are presented in Table 3 and Fig. 1. The metal detection rate ranged from 64.5% of newborn samples for Pb to 100% of both maternal and newborn samples for Ca, Cu, Zn, B, and Hg. Significant differences were shown in all metal concentrations in mothers compared to those in newborns. The median concentrations of essential elements Ca and Zn were higher in newborns’ hair than those observed in their mothers’, while Cu concentrations were lower in the newborns’ hair. On the other hand, the median concentrations of PTM were higher in mothers’ hair than those observed in newborns, except for B and Sb, which were lower. The concentrations of essential elements were found in the following order: Cu < Zn < Ca for mothers and newborns, while PTM concentrations showed this order: Cd < Sb < As < U < Hg < V < Pb < B for mothers’ hair and U < Cd < As < Sb < Pb < Hg < V < B for newborns’ hair.

**Table 3:** Essential elements and PTM concentrations.

Element	Samples with detectable concentrations (%)	Minimum	25% Percentile	Median	75% Percentile	Maximum
Ca *m*, µg/g	100	382.0	791.3	1140.0	1631.0	7837.0
Ca *nb*	100	910.0	1580.0	1892.0	2262.0	4180.0
Cu *m*, µg/g	100	6.0	11.0	12.0	15.0	35.0
Cu *nb*	100	4.0	6.0	7.0	8.0	64.0
Zn *m*, µg/g	100	99.0	158.8	191.0	205.8	1063.0
Zn *nb*	100	96.0	185.3	211.0	235.8	293.0
B *m*, µg/g	100	3.0	5.0	7.0	10.0	21.0
B *nb*	100	5.0	8.0	10.0	19.0	69.0
As *m*, ng/g	88.5	≤0.041	22.0	36.5	54.5	208.0
As *nb*	78.1	≤0.041	4.5	13.0	20.0	123.0
Cd *m*, ng/g	98.9	≤0.069	4.0	7.5	15.5	503.0
Cd *nb*	91.6	≤0.069	2.0	3.0	7.0	332.0
Hg *m*, ng/g	100	21.0	65.2	106.0	209.0	1018.0
Hg *nb*	100	14.0	43.2	76.5	143.5	884.0
Sb *m*, ng/g	100	3.0	12.2	18.0	29.0	2870.0
Sb *nb*	98.9	≤0.060	19.0	34.0	96.0	1320.0
Pb *m*, ng/g	91.6	≤0.065	114.8	223.5	499.3	2746.0
Pb *nb*	64.5	≤0.065	≤ 0.065	54.5	178.0	2686.0
U *m*, ng/g	100	1.0	16.0	38.0	78.75	529.0
U *nb*	94.7	≤0.071	1.0	1.0	4.0	31.0
V *m*, ng/g	100	37	110	176	356.3	917
V *nb*	93.7	≤0.028	41.25	77.5	97	194

*m* mother, *nb* newborn.

### Relationship between each essential element or PTM hair concentration between mothers and newborns

**Fig. 2:**
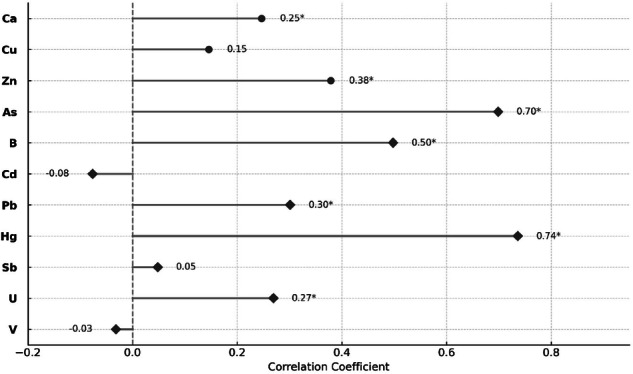
Partial correlation coefficients between maternal and newborn’s essential and PTM concentrations in the hair. Adjusted for mother age, passive smoking, socioeconomic status, frequent use of lead-glazed ceramics, and living near an industry. Circles represent essential elements, and diamonds denote PTM. Sperman’s rank correlation test was used. *p < 0.05.

Partial correlations between maternal and newborn hair concentrations of essential elements and PTM after adjustment by potential confounders are presented in Fig. 2. Among essential elements, significant positive associations were observed for Zn (rho = 0.3786) and Ca (rho = 0.2467), whereas Cu showed a non-significant correlation. In the case of PTM, significant strong correlations were observed for Hg (rho = 0.7365), As (rho = 0.6987), and B (rho = 0.4980), followed by moderate correlations for Pb (rho = 0.3012) and U (rho = 0.2691). In contrast, no significant correlations were found for Cu, Cd, Sb, or V. In exploratory stratified analyses (Table S5), correlations for As and Hg remained consistently strong across all subgroups, whereas several metals showed heterogeneity by exposure-related factors. For example, Zn correlations were higher among mothers with not-low socioeconomic status and in the subgroup classified as not frequent seafood consumption, and Cu became significant only in the non-low socioeconomic status subgroup. Additionally, Sb correlations were evident among passive smokers and participants living near an industry, while Pb and U showed moderate correlations in some subgroups. These stratified results should be interpreted cautiously, given the small sample sizes within subgroups.

### Principal components in metal concentrations of maternal and newborn hair

**Fig. 3:**
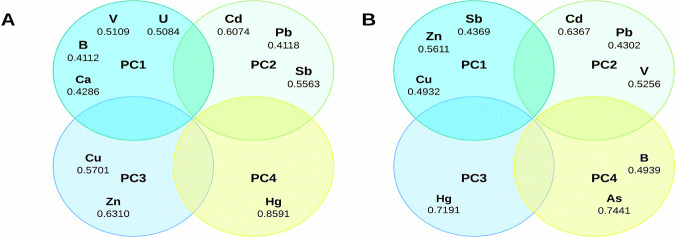
Principal components diagram of metal concentrations. **A** Diagram of four PCs of metal concentrations in mothers’ hair and **B** Diagram of four PCs of metals concentrations in newborns’ hair. The loading factors of each element are presented in their respective PCs.

Results from PCA showed four principal components (PC 1 to 4) in the participants (mother-newborn pairs) suggesting possible common sources to the elements that characterize each main component (Fig. 3). In the case of mothers, PC1 was composed by Ca, B, V and U; PC2 was composed by Cd, Pb and Sb; PC3 by Cu and Zn, and PC4 only by Hg. In the newborns, PC1 was composed of Cu, Sb and Zn; PC2 was composed of Cd, Pb and V; PC3 was composed only of Hg; and PC4 was composed of As and B. To explore more about the relationship between the elements, some positive significant correlations between PTM, essential elements, and PTM-essential elements were observed in the hair of mothers or in the hair of newborns (Tables S6–S7).

## Discussion

In this study, the concentrations and relationships of essential elements and PTM in hair from a population of mother-newborn pairs from the MAMC were evaluated. This is one of the few studies focused on the evaluation of hair metal concentrations as a biomarker of prenatal exposure in a newborn population, and the first in newborns environmentally exposed. Most of the studies evaluate metal exposure in mothers’ samples (blood or urine) as a surrogate of prenatal exposure due to the low accessibility of newborns’ samples [21, 22]. In this sense, hair may be a good biomarker of metal exposure in this development window because hair predicts exposure over a prolonged period (months) and values are consistent with other biomarkers, such as urine and blood [10]. According to ATSDR, hair is usually used to evaluate exposure to As and Hg in unexposed populations, suggesting reference values of ≤ 1.0 µg/g for As and ≤ 200.0 ng/g for Hg [23, 24]. The Cd values in children’s and adults’ hair are ≤ 48.0 ng/g [25]. The median concentrations of As and Cd in mothers’ hair of the present study were within the range of reference values. Unfortunately, there are no reference values for other PTM or essential elements in hair, let alone for newborns.

The concentrations of As, Cd, Hg, Pb, Sb, and U in mothers were within the range of values observed in non-exposed pregnant women reported by several authors [26–29] except for B and V hair concentrations, which were higher. Regarding hair PTM concentrations in newborns, they were within the values observed in non-exposed children under 14 years of age [13, 30], except for B as in their mothers, and interestingly U. The concentrations of B in mothers’ and newborns’ hair in the present study were higher compared to pregnant women from the Russian Federation (up to 22-fold) and children from Spain (up to 5-fold).

A possible source of B and V can be water. In the groundwater from South-central Mexico, B was found 2-fold higher ( > 5000 μg/L) than the WHO guideline (2022), and V concentrations were found 1.7-fold higher (> 381 μg/L) than the ATSDR reference (2013) [31–33]. Boron is a ubiquitous element in soil and water, naturally combined with oxygen (borates), and is recognized as an essential nutrient for plants; fruits, vegetables, pulses, and nuts are the richest sources of B [34]. On the other hand, B can be released into the environment through soil and volatile volcano emanations and deposited mainly in food and drinking water, from anthropogenic activities [35, 36]. Spring water monitoring at five sites near the Popocatepetl volcano (approximately 64 kilometers from the MAMC) from 1994 to 2000 showed B concentrations of around 5000 μg/L, and two of the springs showed concentrations higher than those suggested by the European Community ( < 1000 μg/L) [37]. Unfortunately, B concentrations are not regulated in Mexico. Boron may have positive effects on bone health and mineral metabolism; however, it is not considered an essential element in the human diet [38], and research continues to clarify its role and potential health benefits. There is scarce literature about the human toxicity of B at environmental levels. Some studies have shown negative effects on early growth in humans; Igra et al. (2016) [39] and Hjelm et al. (2019) [40] found negative associations between high levels of B and body size (weight, head circumference, and length) in infants at 0, 3, or 6 months of age.

Regarding V and U, they are naturally present in soil, water, and air, and are released into the environment through industrial activities, vehicle emissions, and volcanic eruptions [31, 41]. In 2016, volcanic ash samples were collected from various locations near the Popocatepetl volcano, and the analysis revealed concentrations of 79.8 µg/g for V and 12.3 µg/g for U [42], suggesting a possible contamination of air and groundwater with both elements.

Concerning the mother-newborn essential elements and PTM relationships, the concentrations of Ca, Zn, As, Hg, Pb, and U were positively correlated, with a wide range of correlation values (rho values from 0.2 to 0.7), indicating a reflection of maternal exposure to their offspring.

Positive correlations between maternal and newborn hair metal concentrations were observed for several potentially toxic metals (PTMs), including Cr, Cs, Hg, Mo, Ni, Sr, U, V, and W, indicating a reflection of maternal exposure in their offspring. In contrast, no such correlations were found for Cu, Cd, Sb and V, suggesting differences in placental transfer efficiencies. These findings were observed among populations in the Gaza war–affected area [43]. Other studies have shown various degrees of correlation between maternal blood and newborn cord blood for essential elements or PTM (correlation coefficients: Hg 0.66, Pb 0.29, Se 0.39) with estimated placental transfer ratios for toxic metals or metalloids ranging from 1.68 (Hg) to 0.18 (Cd). [44]. It is known that PTM uses different ways to cross the placenta. Methyl Hg is actively transported to the placenta by amino acid carriers, Pb freely crosses the placenta by passive diffusion, while metallothionein in the placenta appears to modulate Cd transfer [45].

In general, maternal hair PTM concentrations were higher than those of the newborns, suggesting that the placenta modulates the rate of metal transfer [46]. Interestingly, B and Sb concentrations were 1.4- and 1.6-fold, respectively, higher in newborns’ hair than those observed in their mothers’ hair, suggesting possible accumulation of these PTM in the amniotic fluid, or umbilical cord blood that could be bioavailable during fetal development. Additionally, maternal factors, such as age and BMI, may also modulate metal concentrations in amniotic fluid and maternal serum [47, 48]. In agreement with this, Hjelm et al., and Iwai-Shimada et al. reported that Argentinean and Japanese mothers had higher levels of B (1.26-fold) and Sb (2-fold) in cord blood than in maternal blood [40, 49]. Fetuses can accumulate metals in a different way compared to their mothers, because they have differences in metabolism and unique developmental needs, as was suggested by others [50–53]. Further research is needed to fully understand the implications of these findings on infant health and development.

Concerning the potential contributing sources to PTM and essential elements that were increased in mothers’ or newborns’ hair compared to non-exposed pregnant women and children, such as B, Cd, Pb, V, U, Hg, Ca, and Zn and their relationship between them, the PCA identified four PCs in both mothers and newborns, accounting approximately 60% of the cumulative explained variance, respectively. Particularly, we observed similarities in some PCs, where the same metals (Cd-Pb and Cu-Zn) were present in both the mother (PC2 and PC3, respectively) and the newborn (PC2 and PC1, respectively), suggesting the same source. A potential source of exposure for Cd and Pb is the second-hand tobacco smoke [54], which is consistent with our findings, since passive smoking mothers (35.4%) had higher concentrations of Cd and Pb compared to mothers who were not. Regarding Cu and Zn source, mothers consumed vitamins and supplements during pregnancy, which are necessary for the correct development of the fetus and to avoid short- and long-term complications [1]. Finally, the possible source of Hg, which was found only in a PC in both mothers (PC4) and newborns (PC3), could be through air, since high concentrations of Hg were reported in PM_2.5_ from the Northeast of the MAMC, where the study was conducted [55].

The potential sources of the rest of the metals in PC1, such as V and B, as well as those in PC2 in the mothers, could be air because mothers residing near industrial sites exhibited 2.2-fold higher V levels and 1.4-fold higher B levels than those who did not. Many industrial activities and mobile sources could contribute to PTM exposure, because the air quality in the study area frequently exceeds the annual average PM_2.5_ levels up to 3- to 5-fold the WHO air quality guidelines [56]. High levels of PM_2.5_ and the presence of adsorbed metals, such as Cu, Cd, Cr, Fe, Mn, Ni, Pb, V, Sb, and Zn, have been reported in the Northeast monitoring air station where participants of the present study lived [16, 57]. Furthermore, numerous industrial activities in this area manufacture detergents, which may use B in their production processes [58]. We observed different groups of exposure in the mothers and newborns as shown by the PCA, probably because the placental barrier is more permeable to some xenobiotics and to differences in how PTM pass through due to their physicochemical characteristics [59].

The importance of knowing the exposure to PTM during the intrauterine stage lies in the prevention of adverse effects in childhood. A previous study in newborns, whose mothers were residents of the MAMC, showed altered DNA methylation of *LINE1* and *Nrf2* and *OGG1* genes, which was associated with the interaction between some PTM (As, Hg, Mn, Mo, and Pb) quantified in maternal urine [60]. In addition, PTM concentrations in the cord blood, evaluated as mixtures (as PC), negatively affected the DNA repair capacity, with As being the main contributor to this effect [61]. Maternal Cd concentrations during early pregnancy were significantly associated with birth weight [5], and maternal exposure to Pb and V, individually and as a mixture, was inversely associated with fetal weight at 34-36 weeks of gestation [62]. It would be interesting to evaluate a larger population sample and some post-delivery outcomes in our population, which are limitations of our study.

The results of this study showed the exposure level to PTM in mother-newborn pairs from the highly contaminated MAMC. The concentrations of all PTM, except for B and Sb, were higher in mothers’ hair compared to those in newborns’ hair, and concentrations of all essential elements, except for Cu, were also higher in newborns’ hair, suggesting a regulation of placental transference to the fetus. The relationship between hair concentrations of Ca, Zn, As, B, Hg, Pb, and U in mothers and newborns showed significant positive correlations. However, there were no significant correlations in the concentrations of Cu, Cd, Sb, and V in hair, possibly due to their different transplacental transfer. The presence of PTMs in newborns’ hair suggests that hair could represent a good matrix for biomonitoring prenatal exposures. Finally, this work can be a baseline for future studies conducted in pregnant women or neonates to learn more about prenatal exposures and to contribute to establishing reference values of metal concentrations in these vulnerable populations.

## Supplementary information

Supplementary information

## Data Availability

All data generated and analyzed during this study are included in this published article [and its supplementary information files].
