# Supplementary material for: Hair metal concentrations in a mother-newborn population from a polluted megacity: an indicator of prenatal metal exposure
Source: J Expo Sci Environ Epidemiol. 2026 Apr 11;36(4):788–95. doi: 10.1038/s41370-026-00869-4 (PMC13331732; doi:10.1038/s41370-026-00869-4)
Supplement: Supplementary file 1 — Supplementary information [file 41370_2026_869_MOESM1_ESM.docx]

**Supplementary table 1. Parameters for the ICP-MS**

| Parameter | Setting |
| --- | --- |
| Instrument |  |
|  | 0.8 L min^-1^ Ar |
|  | 1.2 L min ^-1^ Ar |
|  | 18 L min ^-1^ Ar |
|  | 1600 W |
|  | < 1^-8^ torr |
|  |  |
| Timing |  |
| Scan mode | Standard |
| Sweeps/Reading | 20 |
| Readings/Replicate | 1 |
| Replicates | 3 |
| Dwell time | 50 ms |
| Detector mode | Dual |
| Calibration regression type | Simple Linear |
|  |  |
| Method |  |
| Sample Flush | 35 s |
| Read Delay | 15 s |
| Wash | 45 s |

**Supplementary table 2. Hair metal concentrations in mothers (M) and newborns (NB) concerning the consumption of non-nutritive substances and vitamins.**

| Characteristic | Number of subjects | As | | B | | | | Cd | | | Hg | | Sb | | Pb | | U | | V | | Ca | | | | Cu | | Zn | | |  |
| --- | --- | --- | --- | --- | --- | --- | --- | --- | --- | --- | --- | --- | --- | --- | --- | --- | --- | --- | --- | --- | --- | --- | --- | --- | --- | --- | --- | --- | --- | --- |
|  |  | M | NB | | M | NB | M | | NB | M | | NB | M | NB | M | NB | M | NB | M | NB | | M | NB | M | | NB | | M | NB | |
| Consumption of vitamins and minerals |  |  |  | |  |  |  | |  |  | |  |  |  |  |  |  |  |  |  | |  |  |  | |  | |  |  | |
| Folic acid and ferrous sulfate | 28 | 37 | 13 | | 8 | 12 | 8 | | 3 | 111 | | 74 | 17 | 59 | 259 | 70 | 56 | 2 | 264 | 76 | | 1150 | 2036 | 13 | | 8 | | 189 | 214.5 | |
| Folic acid, ferrous sulfate, calcium and B-complex | 50 | 38 | 14 | | 7 | 14 | 6 | | 3 | 106 | | 73 | 18 | 30 | 232 | 50 | 33 | 2 | 156 | 81 | | 1090 | 1867 | 12 | | 7 | | 1911 | 211 | |
| Folic acid | 3 | 11 | 3 | | 7 | 10 | 7 | | 5 | 142 | | 68 | 11 | 86 | 154 | 56 | 21 | 1 | 135 | 55 | | 1534 | 1384 | 11 | | 5 | | 160 | 216 | |
| B-complex | 3 | 51 | 13 | | 6 | 10 | 14 | | 2 | 50 | | 35 | 23 | 26 | 166 | 88 | 29 | 1 | 153 | 77 | | 795 | 1899 | 14 | | 8 | | 170 | 219 | |
| Calcium | 4 | 26 | 2 | | 9 | 15 | 14 | | 2 | 199 | | 141 | 24 | **17*** | 344 | 38 | **1*** | 1 | 195 | 27 | | 1491 | 1836 | 12 | | 6 | | 176 | 180 | |
| No consumption | 4 | 41 | **27*** | | 6 | 9 | 15 | | 2 | 164 | | 116 | 21 | 39 | 368 | 176 | 68 | 1.5 | 233 | 90 | | 1137 | 1911 | 11 | | 8 | | 202 | 229 | |
| Consumption of non-nutritive substances |  |  |  | |  |  |  | |  |  | |  |  |  |  |  |  |  |  |  | |  |  |  | |  | |  |  | |
| Soil or mud | 13 | 48 | 13 | | 7 | 9 | 7 | | 3 | 96 | | 78 | 22 | 42 | 205 | 146 | 35 | 2 | 246 | 86 | | 1114 | 2013 | 11 | | 7 | | 194 | 199 | |
| Chalk | 3 | 43 | 29 | | 11 | 13 | 4 | | 3 | 106 | | 73 | 17 | 191 | 535 | 243 | 72 | 5 | 919 | 71 | | 977 | 1773 | 13 | | 9 | | 201 | 228 | |
| Plaster | 1 | 91 | 25 | | 10 | 36 | 6 | | 6 | 100 | | 289 | 30 | 23 | 144 | 558 | 109 | **8*** | 859 | 158 | | 490 | 3123 | 19 | | 14 | | 155 | 293 | |
| No consumption | 79 | 34 | 13 | | 8 | 13 | 8 | | 3 | 109 | | 75 | 18 | 34 | 227 | 42 | 36 | 1 | 167 | 74 | | 1158 | 1876 | 12 | | 7 | | 191 | 212 | |

Values represent concentrations for essential elements and B in µg/g and PTM in ng/g. Kruskal Wallis test ** p* < 0.05

**Supplementary table 3. Hair metal concentrations in the (M) and newborns (NB) concerning mothers**' **habits.**

| Characteristic | Number of subjects | As | | B | | Cd | | Hg | | Sb | | Pb | | U | | V | | Ca | | Cu | | Zn | |
| --- | --- | --- | --- | --- | --- | --- | --- | --- | --- | --- | --- | --- | --- | --- | --- | --- | --- | --- | --- | --- | --- | --- | --- |
|  |  | M | NB | M | NB | M | NB | M | NB | M | NB | M | NB | M | NB | M | NB | M | NB | M | NB | M | NB |
| Frequent consumption of seafood |  |  |  |  |  |  |  |  |  |  |  |  |  |  |  |  |  |  |  |  |  |  |  |
| Yes | 46 | 39 | 13 | 8 | 13< | 8 | **2*** | 101 | 82 | 19 | 42 | 238 | 31 | 29 | 1 | 175 | 75 | 1031 | 1891 | 12 | 7 | 187 | 202 |
| No | 50 | 34 | 13 | 7 | 12 | 7 | **5** | 115 | 68 | 18 | 30 | 223 | 59 | 47 | 1 | 191 | 77 | 1246 | 1893 | 12 | 7 | 193 | 216 |
| Use of dental amalgams |  |  |  |  |  |  |  |  |  |  |  |  |  |  |  |  |  |  |  |  |  |  |  |
| Yes | 14 | 36 | 15 | 10 | 16 | 5 | 5 | 125 | 96 | 14 | 24 | 215 | 121 | 29 | 1 | 118 | 84 | 1081 | 1742 | 14 | 7 | 199 | 216 |
| No | 80 | 36 | 12 | 7 | 11 | 8 | 3 | 103 | 74 | 20 | 36 | 217 | 52 | 40 | 1 | 207 | 77 | 1142 | 1899 | 12 | 7 | 189 | 204 |
| Frequent use of lead-glazed ceramics |  |  |  |  |  |  |  |  |  |  |  |  |  |  |  |  |  |  |  |  |  |  |  |
| Yes | 29 | 32 | 15 | 7 | 10 | 5 | 3 | **165*** | 93 | 17 | 29 | 296 | 69 | 57 | 1 | 188 | 63 | 1008 | 1763 | 13 | 7 | 187 | 213 |
| No | 67 | 38 | 12 | 8 | 13 | 8 | 3 | **98** | 72 | 18 | 34 | 206 | 53 | 34 | 2 | 172 | 80 | 1204 | 1938 | 12 | 7 | 193 | 208 |
| Passive smoker |  |  |  |  |  |  |  |  |  |  |  |  |  |  |  |  |  |  |  |  |  |  |  |
| Yes | 34 | 42 | 14 | 8 | 10 | 7 | **3**** | 112 | 74 | 20 | 33 | 232 | **79*** | 42 | **2*** | 215 | 80 | 1095 | 1815 | 12 | 7 | 187 | 216 |
| No | 62 | 29 | 12 | 7 | 14 | 7 | **2** | 104 | 91 | 17 | 35 | 213 | **22** | 31 | **1** | 139 | 71 | 1343 | 2001 | 13 | 7 | 193 | 196 |

Essential elements and B in µg/g. and PTM were reported in ng/g. U Man-Whitney test ** p* < 0.05, *** p* < 0.01

**Supplementary table 4. Hair metal concentrations in the mothers (M) and newborns (NB) concerning sources of metal exposure near the mothers´ home.**

| Characteristic | Number of subjects | | As | | | | B | | | | Cd | | | | Hg | | | | Sb | | Pb | | U | | | V | | | | Ca | | | | Cu | | | | Zn | | |
| --- | --- | --- | --- | --- | --- | --- | --- | --- | --- | --- | --- | --- | --- | --- | --- | --- | --- | --- | --- | --- | --- | --- | --- | --- | --- | --- | --- | --- | --- | --- | --- | --- | --- | --- | --- | --- | --- | --- | --- | --- |
|  |  | | M | | NB | | M | | NB | | M | | NB | | M | | NB | | M | NB | M | NB | M | NB | | M | | NB | | M | | NB | | M | | NB | | M | | NB |
| Shops at home |  | |  | |  | |  | |  | |  | |  | |  | |  | |  |  |  |  |  |  | |  | |  | |  | |  | |  | |  | |  | |  |
| Yes | 11 | | 30 | | 13 | | 8 | | 9 | | 7 | | 2 | | 133 | | 93 | | 17 | 34 | 405 | 47 | 75 | 2 | | 217 | | 41 | | 1281 | | 1884 | | 12 | | 7 | | **139** | | 186 |
| No | 85 | | 38 | | 13 | | 7 | | 13 | | 8 | | 3 | | 103 | | 74 | | 18 | 34 | 220 | 55 | 35 | 1 | | 176 | | 78 | | 1133 | | 1899 | | 12 | | 7 | | **193*** | | 212 |
| Shops near the home |  | |  | |  | |  | |  | |  | |  | |  | |  | |  |  |  |  |  |  | |  | |  | |  | |  | |  | |  | |  | |  |
| Blacksmith shop |  | |  | |  | |  | |  | |  | |  | |  | |  | |  |  |  |  |  |  | |  | |  | |  | |  | |  | |  | |  | |  |
| Yes | 19 | | 39 | | 13 | | 7 | | 9 | | 7 | | 4 | | 125 | | 100 | | 16 | 36 | 186 | 87 | **57*** | **3*** | | 267 | | 90 | | 1281 | | 1977 | | 13 | | 7 | | 195 | | 222 |
| No | 77 | | 34 | | 13 | | 8 | | 13 | | 8 | | 3 | | 100 | | 65 | | 19 | 33 | 227 | 52 | **32** | **1** | | 169 | | 73 | | 1127 | | 1884 | | 12 | | 7 | | 187 | | 210 |
| Shoe repair shop |  | |  | |  | |  | |  | |  | |  | |  | |  | |  |  |  |  |  |  | |  | |  | |  | |  | |  | |  | |  | |  |
| Yes | 8 | | 35 | | 9 | | 6.5 | | 9 | | 7 | | 5 | | 108 | | 68 | | 22 | 102 | 267 | 23 | 49 | 1 | | 194 | | 98 | | 1254 | | 1514 | | 13 | | 8 | | 179 | | 192 |
| No | 88 | | 36 | | 13 | | 8 | | 13 | | 8 | | 3 | | 106 | | 81 | | 18 | 33 | 218 | 55 | 35 | 1 | | 176 | | 73 | | 1130 | | 1915 | | 12 | | 7 | | 191 | | 212 |
| Tire repairshop |  | |  | |  | |  | |  | |  | |  | |  | |  | |  |  |  |  |  |  | |  | |  | |  | |  | |  | |  | |  | |  |
| Yes | 10 | | 21 | | 8 | | 8.5 | | 9 | | 9 | | 2 | | 99 | | 64 | | 16 | 78 | 208 | 80 | 40 | 1 | | 218 | | 58 | | 1391 | | 1851 | | 12 | | 7 | | 173 | | 192 |
| No | 86 | | 37 | | 14 | | 8 | | 13 | | 7 | | 3 | | 107 | | 81 | | 18 | 32 | 223 | 54 | 38 | 1 | | 176 | | 78 | | 1130 | | 1915 | | 12 | | 7 | | 191 | | 212 |
| Vehicle repair shop |  | |  | |  | |  | |  | |  | |  | |  | |  | |  |  |  |  |  |  | |  | |  | |  | |  | |  | |  | |  | |  |
| Yes | 25 | | 29 | | 12 | | 7 | | 10 | | 5 | | 4 | | 102 | | 61 | | 15 | 50 | 186 | 69 | 46 | 2 | | 207 | | 74 | | 1061 | | 1807 | | 13 | | 7 | | 179 | | 201 |
| No | 71 | | 38 | | 14 | | 8 | | 13 | | 8 | | 3 | | 106 | | 84 | | 22 | 33 | 237 | 54 | 35 | 1 | | 176 | | 78 | | 1181 | | 1955 | | 12 | | 7 | | 193 | | 212 |
| Industry |  | |  | |  | |  | |  | |  | |  | |  | |  | |  |  |  |  |  |  | |  | |  | |  | |  | |  | |  | |  | |  |
| Yes | 18 | | 49 | | 15 | | **10*** | | 15 | | 11 | | 2 | | 148 | | 84 | | 19 | 26 | 257 | 70 | 56 | 1 | | **325**** | | 61 | | 1427 | | 2109 | | 12 | | 7 | | 193 | | 222 |
| No | 76 | | 33 | | 12 | | **7** | | 10 | | 7 | | 3 | | 103 | | 79 | | 18 | 36 | 218 | 46 | 30 | 2 | | **146** | | 79 | | 1135 | | 1857 | | 12 | | 7 | | 190 | | 205 |
| Family member with employment related to some source of exposure | |  | |  | |  | |  | |  | |  | |  | |  | |  |  |  |  |  |  |  |  | |  | |  | |  | |  | |  | |  | |  | |
| Yes | | 41 | | 32 | | 14 | | 8 | | 13 | | 8 | | 2 | | 97 | | 82 | 18 | 29 | 227 | 17 | 46 | 1 | 164 | | 78 | | 1023 | | 1910 | | 12 | | 7 | | 191 | | 216 | |
| No | | 55 | | 38 | | 12 | | 7 | | 10 | | 7 | | 3 | | 107 | | 76 | 21 | 53 | 234 | 59 | 34 | 1 | 212 | | 78 | | 1248 | | 1871 | | 12 | | 7 | | 191 | | 205 | |

Essential elements and B in µg/g. and PTM were reported in ng/g. U Man-Whitney test * p < 0.05, ** p < 0.01

**Supplementary table 5. Stratified partial correlations between maternal and newborns´ hair metal concentrations.**

| **Element** | **Frequent use of lead-glazed ceramics** | | **Passive smoking** | | **Socioeconomic Status** | | **Living near an industry** | | **Frequent consumption of seafood** | |
| --- | --- | --- | --- | --- | --- | --- | --- | --- | --- | --- |
|  | Yes  (n=28) | No  (n=65) | Yes  (n=33) | No  (n=60) | Low  (n=40) | Not low  (n=53) | Yes  (n=18) | No  (n=75) | Yes  (n=44) | No  (n=49) |
| **Ca** | **0.4314*** | 0.2422 | 0.1662 | **0.2805*** | 0.2477 | 0.2655 | 0.4134 | **0.2840*** | 0.2315 | 0.1585 |
| **Cu** | **0.5532*** | 0.0264 | 0.1782 | 0.1075 | -0.0110 | **0.3680*** | -0.0379 | 0.2029 | 0.1879 | 0.1143 |
| **Zn** | **0.4598*** | **0.2980*** | 0.1631 | **0.4132*** | 0.2610 | **0.4658**** | -0.4414 | **0.4545**** | 0.2594 | **0.6150**** |
| **As** | **0.6726**** | **0.7045**** | **0.6192**** | **0.7546**** | **0.7003**** | **0.7085**** | **0.5939*** | **0.7187**** | **0.6644**** | **0.7450**** |
| **B** | 0.3299 | **0.6324**** | **0.5402*** | **0.5129**** | **0.5679**** | **0.4866**** | **0.5242*** | **0.4883**** | **0.6282**** | **0.4009*** |
| **Cd** | -0.2906 | -0.0460 | -0.2030 | -0.0836 | 0.0345 | -0.1594 | -0.1391 | -0.0824 | -0.1699 | 0.0591 |
| **Pb** | 0.2790 | **0.3285*** | **0.4368*** | 0.2508 | 0.1265 | **0.4159*** | 0.1683 | **0.3347*** | 0.0093 | **0.4289*** |
| **Hg** | **0.8232**** | **0.7001**** | **0.6572**** | **0.7672**** | **0.6577**** | **0.7725**** | **0.6572*** | **0.7400**** | **0.7271**** | **0.7713**** |
| **Sb** | -0.0122 | 0.0657 | **0.4657*** | -0.2050 | -0.1851 | 0.1759 | **0.5944*** | -0.1443 | -0.0367 | 0.0760 |
| **U** | **0.3900*** | 0.2213 | 0.0934 | **0.3684*** | 0.2506 | **0.3230*** | 0.3398 | 0.2258 | 0.3981* | 0.1799 |
| **V** | 0.1571 | -0.1532 | 0.0607 | -0.0164 | 0.0691 | -0.1074 | -0.1938 | 0.0828 | -0.1151 | -0.1497 |

Residuals were obtained from linear regression models adjusting for mother age, passive smoking, socioeconomic status, frequent use of lead-glazed ceramics, and living near an industry; the stratification variable was excluded from the adjusted model in each analysis. ** p<*0.05, *** p* < 0.01

**Supplementary table 6. Relationship between the concentrations of metals in the mothers´ hair.**

|  | **As** | **B** | **Cd** | **Hg** | **Sb** | **Pb** | **V** | **U** | **Ca** | **Cu** | **Zn** |
| --- | --- | --- | --- | --- | --- | --- | --- | --- | --- | --- | --- |
| **As** | 1 |  |  |  |  |  |  |  |  |  |  |
| **B** | 0.3175* | 1 |  |  |  |  |  |  |  |  |  |
| **Cd** | 0.3042* | 0.1542 | 1 |  |  |  |  |  |  |  |  |
| **Hg** | -0.1003 | -0.0931 | 0.0607 | 1 |  |  |  |  |  |  |  |
| **Sb** | 0.2786* | 0.0403 | 0.6055** | 0.0676 | 1 |  |  |  |  |  |  |
| **Pb** | **0.3876**** | 0.2225* | **0.4618**** | 0.1047 | 0.3428** | 1 |  |  |  |  |  |
| **V** | 0.3157* | 0.2821* | **0.3055*** | 0.0551 | 0.2300* | 0.2465* | 1 |  |  |  |  |
| **U** | 0.3241* | 0.3004* | 0.2698* | 0.0068 | 0.1430 | **0.3856**** | **0.5181**** | 1 |  |  |  |
| **Ca** | -0.0960 | 0.1360 | 0.1258 | 0.1140 | -0.0585 | 0.0625 | 0.5070** | **0.2743*** | 1 |  |  |
| **Cu** | 0.0679 | 0.0971 | 0.2124* | 0.0400 | 0.0774 | 0.3167* | 0.1643 | 0.2099* | 0.1284 | 1 |  |
| **Zn** | 0.0332 | -0.1039 | -0.0471 | -0.0343 | -0.1492 | -0.0033 | 0.0716 | 0.0598 | **0.3705**** | **0.2215*** | 1 |

Spearman's rank correlation. ** p<*0.05, *** p* < 0.01

**Supplementary table 7. Relationship between the concentrations of metals in the newborns´ hair.**

|  | **As** | **B** | **Cd** | **Hg** | **Sb** | **Pb** | **V** | **U** | **Ca** | **Cu** | **Zn** |
| --- | --- | --- | --- | --- | --- | --- | --- | --- | --- | --- | --- |
| **As** | 1 |  |  |  |  |  |  |  |  |  |  |
| **B** | 0.1614 | 1 |  |  |  |  |  |  |  |  |  |
| **Cd** | 0.1128 | 0.0872 | 1 |  |  |  |  |  |  |  |  |
| **Hg** | -0.0472 | 0.1214 | 0.0714 | 1 |  |  |  |  |  |  |  |
| **Sb** | 0.0575 | -0.0714 | 0.0977 | -0.1009 | 1 |  |  |  |  |  |  |
| **Pb** | **0.3469**** | -0.0150 | **0.2818*** | 0.0989 | 0.0237 | 1 |  |  |  |  |  |
| **V** | 0.0436 | -0.2522 | **0.3887**** | -0.0927 | 0.1110 | 0.1895 | 1 |  |  |  |  |
| **U** | 0.1515 | -0.1534 | 0.0511 | -0.0006 | 0.0472 | **0.2724*** | **0.4821**** | 1 |  |  |  |
| **Ca** | 0.1793 | 0.2957* | -0.1430 | 0.2410* | -0.0046 | 0.1490 | -0.0302 | **0.2875*** | 1 |  |  |
| **Cu** | 0.1679 | 0.1203 | -0.0149 | -0.1144 | 0.0381 | 0.1593 | 0.038 | 0.2744 | 0.1049 | 1 |  |
| **Zn** | 0.4573** | -0.0366 | 0.0213 | -0.0887 | 0.1392 | 0.3881** | 0.1453 | 0.3085* | **0.2824*** | **0.2596*** | 1 |

Spearman's rank correlation. ** p<*0.05, *** p* < 0.01

**B**

**A**

**Supplementary figure 1. Scree plot of eigenvalues after Principal Components Analysis performed with the metals quantified in the mother's (A) and newborn’s (B) hair.** The red line represents the cut-off point for selecting the number of principal components.


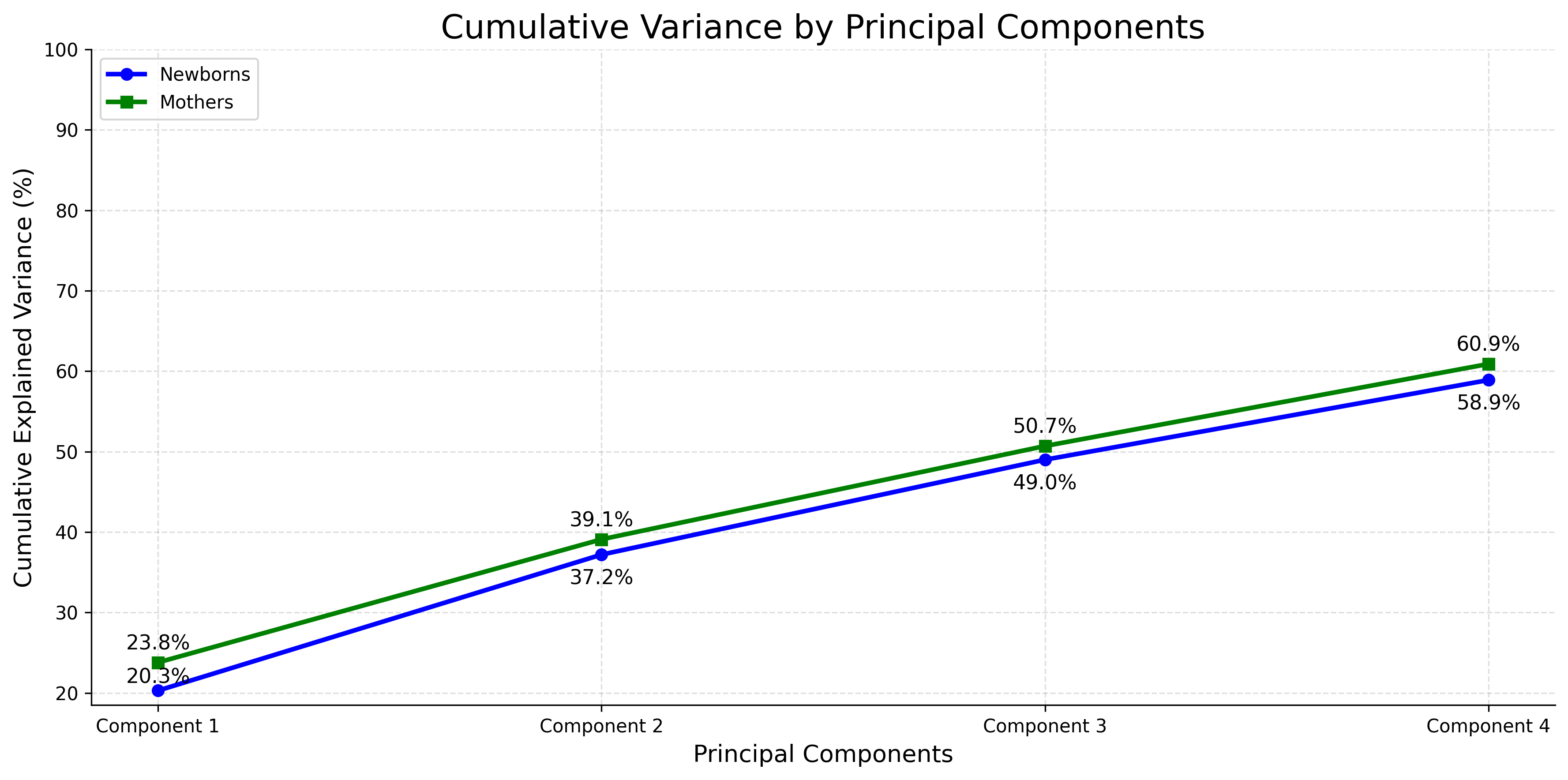
**Supplementary figure 2. Cumulative variance explained (%) by the first four principal components in maternal and newborn datasets.** PCA was performed on log-transformed metal concentrations. Newborn data are represented with blue circles; maternal data are represented with green squares.
